# State of the Art and New Concepts in Giant Cell Tumor of Bone: Imaging Features and Tumor Characteristics

**DOI:** 10.3390/cancers13246298

**Published:** 2021-12-15

**Authors:** Anna Parmeggiani, Marco Miceli, Costantino Errani, Giancarlo Facchini

**Affiliations:** 1 Diagnostic and Interventional Radiology Unit, IRCCS Istituto Ortopedico Rizzoli, Via Pupilli 1, 40136 Bologna, Italy; marco.miceli@ior.it (M.M.); giancarlo.facchini@ior.it (G.F.); 2 Department of Orthopaedic Oncology, IRCCS Istituto Ortopedico Rizzoli, Via Pupilli 1, 40136 Bologna, Italy; costantino.errani@ior.it

**Keywords:** giant cell tumor of bone, imaging, radiology, radiomics, surgery, prognosis, recurrence

## Abstract

**Simple Summary:**

The 2020 World Health Organization classification of soft tissue and bone tumors classified the giant cell tumor of bone (GCTB) as an intermediate malignant tumor, with locally aggressive behavior and high recurrence rate. Imaging plays a pivotal role in the assessment of GCTB, and this review tries to summarize the main concepts about GCTB histopathogenesis and new biomarkers, describing those GCTB imaging findings which could possibly be explained by tumor molecular alterations. We have illustrated pre-operative imaging features related to prognosis and radiological findings for response evaluation after surgical treatment and denosumab administration. We have also reported the results described in literature regarding the role of radiomics in aiding GCTB diagnosis, predicting possible post-treatment recurrence and providing a quantitative assessment of the response to denosumab treatment.

**Abstract:**

Giant cell tumor of bone (GCTB) is classified as an intermediate malignant tumor due to its locally aggressive behavior, burdened by high local recurrence rate. GCTB accounts for about 4–5% of all primary bone tumors and typically arises in the metaphysis and epiphyses of the long tubular bones. Mutation of gene H3F3A is at the basis of GCTB etiopathogenesis, and its immunohistochemical expression is a valuable method for practical diagnosis, even if new biomarkers have been identified for early diagnosis and for potential tumor recurrence prediction. In the era of computer-aided diagnosis, imaging plays a key role in the assessment of GCTB for surgical planning, patients’ prognosis prediction and post treatment evaluation. Cystic changes, penetrating irregular margins and adjacent soft tissue invasion on preoperative Magnetic Resonance Imaging (MRI) have been associated with a higher rate of local recurrence. Distance from the tumor edge to the articular surface and thickness of unaffected cortical bone around the tumor should be evaluated on Computed Tomography (CT) as related to local recurrence. Main features associated with local recurrence after curettage are bone resorption around the graft or cement, soft tissue mass formation and expansile destruction of bone. A denosumab positive response is represented by a peripherical well-defined osteosclerosis around the lesion and intralesional ossification. Radiomics has proved to offer a valuable contribution in aiding GCTB pre-operative diagnosis through clinical-radiomics models based on CT scans and multiparametric MR imaging, possibly guiding the choice of a patient-tailored treatment. Moreover, radiomics models based on texture analysis demonstrated to be a promising alternative solution for the assessment of GCTB response to denosumab both on conventional radiography and CT since the quantitative variation of some radiomics features after therapy has been correlated with tumor response, suggesting they might facilitate disease monitoring during post-denosumab surveillance.

## 1. Introduction

According to the 2020 World Health Organization classification of soft tissue and bone tumors, giant cell tumor of bone (GCTB) is classified as an intermediate malignant tumor, due to its locally aggressive behavior but low metastatic tendency, usually with pulmonary spread [1,2]. GCTB is an osteoclastic giant cell-rich tumor composed of neoplastic mononuclear stromal cells with a monotonous appearance admixed with macrophages and osteoclast-like giant cells [2].

GCTB accounts for about 4–5% of all primary bone tumors and typically arises in patients aged between 20 and 40 years, with a slight prevalence in females (female to male ratio of 1.2:1) [1,3]. Its incidence rate is challenging to quantify due to the lack of population-level statistics in many countries, and it is approximately estimated at about 1.2–1.7 cases per 1 million inhabitants per year [4,5,6,7].

The main GCTB onset locations are the metaphysis and epiphyses of the long tubular bones, including the distal femur (26%), proximal tibia (20%) and distal radius (11%), while it less frequently affects the sacrum, spine, pelvis and short tubular bones of hands and feet [8,9,10].

Multicentric GCTB is extremely unusual, and it is distinguished into synchronous, when multiple lesions arise within an interval of 6 months, or metachronous, when the second lesion appears 6 months after the initial GCTB [9]. It usually affects younger patients (about 21 years of age) and more often manifests as synchronous lesions [9,11].

Malignant GCTB occurs in a small percentage of cases, and it is associated with poor prognosis and sarcomatous pulmonary metastases [12,13]. It is classified in primary malignant GCTB (PMGCTB) or secondary malignant GCTB (SMGCTB) whether or not it arises at the site of a recurrent lesion previously diagnosed as typical GCTB [14]. SMGCTBs are more frequent than PMGCTBs, and they typically develop after radiotherapy, the most common form, or after surgery [13,15,16]. Moreover, late local recurrence of GCTB, intended as a local recurrence after 2 years or more, has been proven to be a prognostic factor for unfavorable malignant transformation [12,17].

Due to its locally aggressive behavior, GCTB clinical presentation often includes pain, swelling and mobility impairment of the involved joint [18]. The painful symptomatology is due to tumor growth, which induces expansion of the periosteum [8]. The presence of sudden acute pain is usually caused by a pathologic fracture, which is found at diagnosis in almost 10–12% of patients [16,19].

GCTBs arising in the vertebral column are often responsible for back pain and may invade the epidural space, causing spinal cord compression and neurologic symptoms [10]. When affecting the sacrum, GCTB might remain silent in its initial stages, with insidious progressive symptoms evolving over a period of several months, causing misdiagnosis or diagnostic delay, resulting in a large lesion size at initial presentation [20].

The first-line approach for GCTB is surgical removal, where curettage is the most common surgical treatment aimed to remove the tumor and preserve as much bone as possible in order to guarantee good functional outcomes [18,21]. In 2013, the United States Food and Drug Administration approved the use of denosumab as a target therapy for GCTB in patients where surgery is not possible or likely to result in severe morbidity [22]. Denosumab is a fully human monoclonal antibody that inhibits the receptor activation of nuclear factor-kappa B (RANK inhibitor), blocking the osteoclasts and promoting new bone deposition, showing a downstaging effect to less invasive surgery [23,24,25]. However, denosumab administration before curettage has also been associated with a higher recurrence rate and malignant transformation [26,27,28,29,30].

## 2. GCTB Cytology, Histopathogenesis and New Biomarkers in Molecular Targeted Therapy Era

### 2.1. GCTB Cytology

Conventional GCTB is constituted by three types of cells: mononuclear stromal cells, mononuclear histiocytic cells, with the role of macrophage-like cells, and osteoclast-like multinucleated giant cells [2,31]. GCTB fine-needle aspiration usually demonstrates mononucleated stromal cells, disseminated in sheets, perivascular clusters (or, more rarely, single) and multinucleated giant cells adhering to the periphery of mononucleated spindle cells clusters [32].

Stromal cells usually show an oval to elongated fusiform hyperchromatic nuclei, sometimes eccentrically placed, with a well-defined nuclear membrane and evenly distributed chromatin [33,34]. Multinucleated giant cells nuclei are larger, round to oval in shape, with a well-defined nuclear membrane, finely dispersed chromatin and a variable number of nucleoli, from 20 to more than 50 [35,36,37].

### 2.2. GCTB Hystology and Pathogenesis

Mononuclear neoplastic stromal cells be detected by osteoblast-related markers, such as RUNX2 and p63, while mononuclear histiocytic cells and osteoclast-like multinucleated giant cells are positive for CD68 [38].

Stromal cells represent the neoplastic population of the tumor and, in 95% of GCTBs, they present a specific driver mutation of the gene H3F3A on chromosome 1, which, together with the gene H3F3B on chromosome 17, encode histone H3.3, a protein involved in the epigenetic regulation of DNA expression [2,38]. Particularly, approximately 90% of H3F3A mutations lead to substitution of glycine 34 to tryptophan or, more rarely, to leucine [39]. Nevertheless, this and other histone mutations are observed in other sarcomas, where the mutated histones, also called “oncohistones”, produce dysregulation at different levels of epigenetic control [40]. In this regard, G34W mutation immunohistochemical (IHC) expression evaluation is a sensitive, user-friendly and valuable method for practical diagnosis, and it is also very useful for differential diagnosis in giant cell-rich tumors, such as giant cell reparative granuloma, brown tumor of hyperparathyroidism, aneurysmal bone cyst, chondroblastoma and giant cell-rich osteosarcoma, since G34W mutation is extremely rare in these cases [41,42].

It has been reported in the literature that the H3.3-G34W mutation in neoplastic stromal cells is associated with a series of epigenetic alterations that play an important role in the phenotypes of GCTB, such as stochastic genomic instability and increased osteolysis, since H3.3-G34W-associated methylome changes affect the expression of the receptor activator of NF-κB ligand (RANKL) and osteoprotegerin (OPG), the main molecules involved in bone metabolism [43,44]. Particularly, G34W mutation not only promotes bone destruction, but plays a key role in the maintenance of proliferating neoplastic osteoprogenitors through the secretion of factors that recruit osteoclasts within the tumor microenvironment [39]. Neoplastic stromal cells express RANKL, a surface marker, which has a pivotal role in the signaling pathway of bone remodeling and in the differentiation of precursors into giant multinucleated osteoclast-like cells, which can be considered activated components, influencing osteoclastogenesis and cell proliferation (tumorigenesis) [2,22,41]. RANKL overexpression in the tumoral stroma is associated to OPG negative feedback inhibition and decreased OPG gene expression, whose combination induces osteoclastic giant cell formation by the fusion of macrophagic precursors [41,45]. Furthermore, multinucleated giant cells, stromal cells and monocytic cells produce high levels of matrix metalloproteinase 14 (MMP14), which is responsible for the cleavage of membrane-bound RANKL into soluble RANKL, thus promoting osteoclastogenesis [45,46].

The increased osteoclast activity causes bone resorption and tumor cell proliferation responsible for the aggressive osteolytic nature of GCTB [22]. The finding of this signaling pathway in the pathogenesis of GCTB has opened the way to the study of molecules that act as RANK inhibitors, such as denosumab, a human monoclonal antibody which binds to RANK, blocking the osteolytic process, stopping tumor growth and invasion and eventually restoring the density of the remaining bone [47].

A study by Metovic et al. on a series of 50 GCTB has demonstrated an association between programmed death-ligand 1 (PD-L1) immune expression in GCTB and a higher risk of recurrence in terms of disease-free interval, suggesting to perform a PD-L1 immunohistochemical evaluation at diagnosis to select GCTB patients who may potentially be considered for anti-PD-1/PD-L1 therapies [48].

Lübbehüsen et al. [49] observed higher Wee1 pathway activation in GCTB, with consequent overexpression of Wee1, Cdk1 and H3K36me3 in the GCTB tissue samples, potentially serving as biomarkers for a definition of patients with GCTB eligible for a Wee1 inhibition-based therapy. Specifically, a Wee1-kinase inhibitor (MK-1775) proved to be a valuable cytostatic agent to use in GCTB treatment, alone or in combination with chemotherapeutic substances, such as gemcitabine.

Similarly, the group of Chen et al. [50,51] assessed differentially expressed genes in the recurrent GCTB through microarray and IHC, identifying four genes with a statistically significant association with GCTB recurrence: mouse double minute 2 homolog (MDM2) (*p* = 0012), insulin-like growth factor 1 (IGF1) (*p* = 0033), signal transducer and activator of transcription 1 (STAT1) (*p* = 0026) and Rac family small GTPase 1 (RAC1) (*p* = 0007), suggesting they might be used as biomarkers for GCTB recurrence.

As stated by a literature review by Palmini and Brandi [47], many recent studies evaluated the role of micro-RNAs in the pathogenesis of GCTB, describing how some miRNAs can act as tumor suppressors or oncogenes, targeting various genes, such as homeobox A1 (HOXA1), tartrate-resistant acid phosphatase (TRAP), cathepsin K (CK), matrix metallopeptidase 9 (MMP-9), cytochrome C oxidase assembly factor 1 homolog (COA1), protein disulfide isomerase family A member 6 (PDIA6) genes and even the Akt signaling pathway.

Jiang et al. [52] assessed the differential expression of long-chain non-coding RNAs (lncRNAs) in 20 cases of PGCTB, 20 cases of recurrent GCTB and 20 cases of bone trauma tissue, demonstrating that the different expression levels of two lncRNAs (AK124776 and RP11-160A10.2) had valuable roles as predictive biomarkers. Specifically, the expression of AK124776 in bone tissue and serum of patients in the recurrent group was significantly higher than that of the initial group and the normal group, while the expression level of RP11-160A10.2 in the recurrent group was significantly lower than that in the initial group, and the normal group was the highest, with a statistically significant difference (*p* < 0.05). Moreover, according to the receiver operating curve (ROC), the accuracy of AK124776 and RP11-160A10.2 in the diagnosis of giant cell tumor of bone was 0.865 and 0.877, respectively; the accuracy of predicting the recurrence of giant cell tumor of bone was 0.832 and 0.841, respectively, suggesting they can be used as markers for early diagnosis and prediction of tumor recurrence [52].

### 2.3. Malignant GCTB Histology and Pathogenesis

In PMGCTB, an IHC analysis demonstrates a coexistence of conventional GCTB features and a malignant element, such as fibrosarcoma or osteosarcoma components with a transitional zone from the GCTB to the malignant area, where the number of giant cells decrease and the number of mononuclear cells increase [14].

As stated before, SMGCTB involves sarcomatous transformation of a previously treated benign GCTB lesion, and it is commonly associated with prior radiation therapy [53]. Histologically, it corresponds to osteosarcoma, fibrosarcoma or, less frequently, undifferentiated pleomorphic sarcoma [2,38].

Even though mechanisms of malignant changes of GCTB remain unclear, in most cases, a conventional and malignant GCTB shares the H3F3A G34W mutation, suggesting it is preserved even after the malignant transformation [38,54]. However, in the literature, there are reports of some cases of MGCTB where the H3F3A G34W mutation was absent at IHC and molecular evaluation due to the deletion of one of the two alleles of the H3F3A gene, causing an H3F3 mutation-negative progression tumor pathway [55].

The malignant transformation of GCTB has also been associated with TP53 gene mutation and Glutathione peroxidase-1 (GPX-1), an antioxidant enzyme involved in redox signaling, whose transcription is induced by p53 [2,56]. Okubo et al. observed a positive correlation between a high p53 and GPX-1 expression in malignant GCTB (*p* = 0.042) and found that patients with a high GPX-1 expression were at greater risk for early relapse (*p* = 0.003) [56].

## 3. Imaging Contribution in GCTB Management

### 3.1. Imaging Features

The typical radiographic features of GCTB include a purely osteolytic lesion with bone remodeling, characterized by cortical thinning and endosteal scalloping, while periosteal reaction is relatively unusual [57,58]. A periosteal reaction or a Codman’s triangle generally indicates a pathological fracture [59].

**Figure 1:**
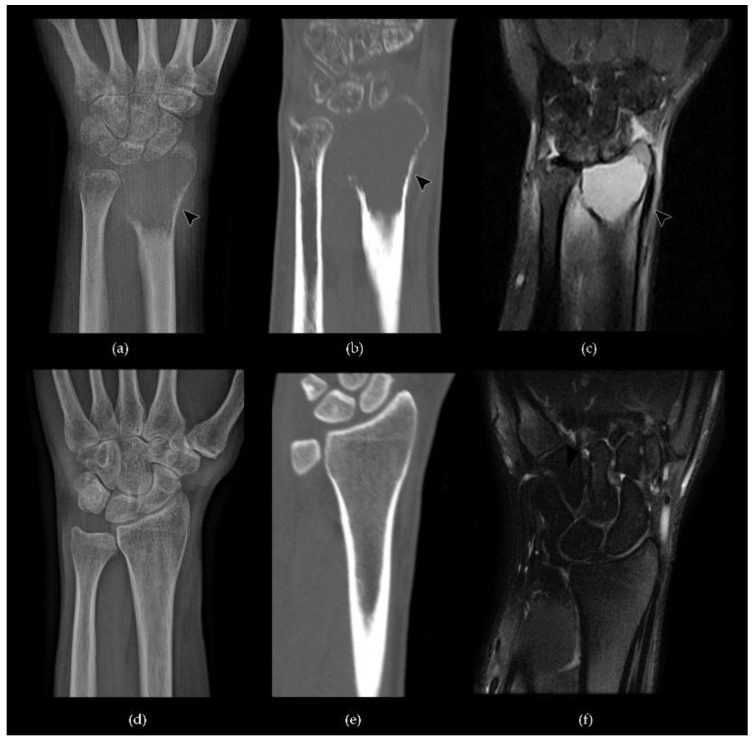
A case of GCTB of the distal metaepiphysis of the right radius with the involvement of the radiocarpal joint (**a**–**c**, arrowhead) compared to normal bone (**d**–**f**): (**a**) conventional radiography shows an osteolytic lesion with cortical thinning and endosteal scalloping with no periosteal reaction; (**b**) a coronal computed tomography (CT) scan highlights multiple cortical interruptions; (**c**) a coronal STIR magnetic resonance imaging (MRI) sequence shows a lesion with homogeneous signal hyperintensity; (**d**–**f**) conventional radiography, CT and STIR MR imaging of normal bone findings.

**Figure 2:**
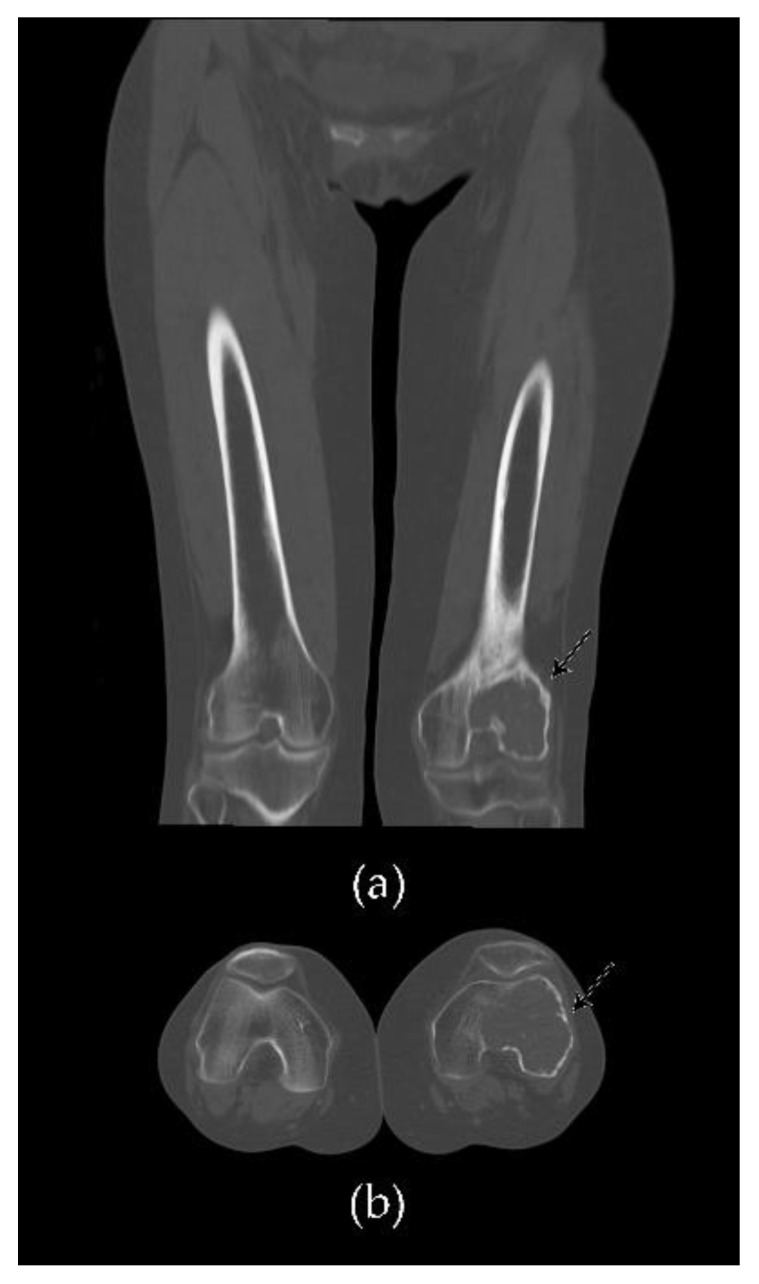
Comparative CT scan of a case of GCTB of the lateral condyle of the left femur: (**a**) coronal and (**b**) axial views demonstrate an extensive osteolytic lesion with cortical thinning (arrows). Note the osteo-structural alteration with a loss of bone trabeculation in comparison with the contralateral right normal limb.

Non-aggressive tumors present a prominent trabeculation with a resultant multiloculated appearance, while aggressive tumors lose the classic trabeculation and are associated with cortex destruction and soft-tissue extension [13,60]. These characteristics can be better evaluated by computed tomography (CT), which has the important advantages of a superior delineation of cortical alterations, which might be invisible on radiographs, and enables a multiplanar evaluation [61]. After contrast medium intravenous administration, mild enhancement of the solid component may occur, while necrotic and cystic zones occasionally show little or no enhancement [62] (Figure 1 and Figure 2).

On magnetic resonance imaging (MRI), GCTB mostly shows low/intermediate signal in T1-weighted images and high signal in T2-weighted images that can be variably inhomogeneous due to the fibrous components, cystic parts as well as the deposition of hemosiderin within the tumor [62,63]. Hemosidenin foci represent a hyperaccumulation of iron due to extravasated erythrocytes phagocytosis from both mononuclear stromal cells and multinucleated giant cells [64].

**Figure 3:**
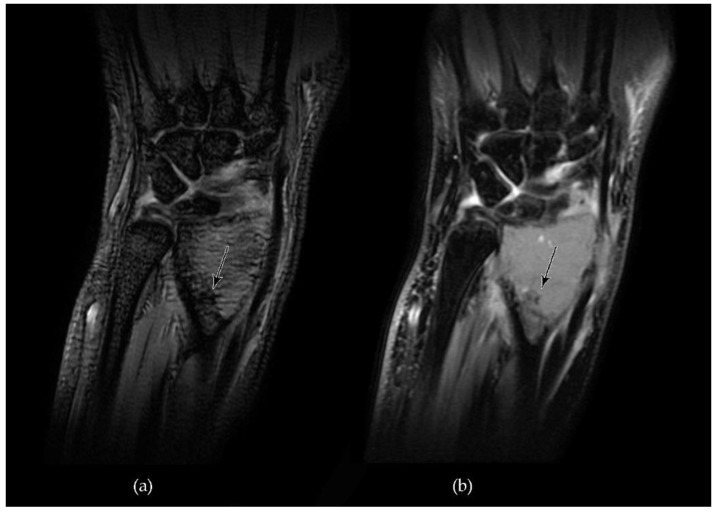
MRI appearance of hemosiderin deposits (arrows) in a case of GCTB of the left radial metaepiphysis (**a**). T2*-W GRE sequence demonstrates a markedly and distinctly hypointense area that appears more tenuously hypointense in a T2-W Fast Spin Echo (FSE) sequence (**b**).

Hemosiderin deposits demonstrate low signal intensity both in T1- and T2-weighted spin echo sequences (T1- and T2-W SE) but can be better identified through the use of T2 star-weighted gradient-echo sequences (T2*-W GRE), where micro-hemorrhages appear as small sharply hypointense foci [64,65]. T2*-W GRE is a particular resonance sequence that found its first main application in the detection of hemorrhagic brain lesions, since it is more sensitive in identifying hemosiderin deposits resulting from microbleeds, which appear as strongly hypointense areas in comparison to T2-W SE sequences [66,67,68] (Figure 3).

**Figure 4:**
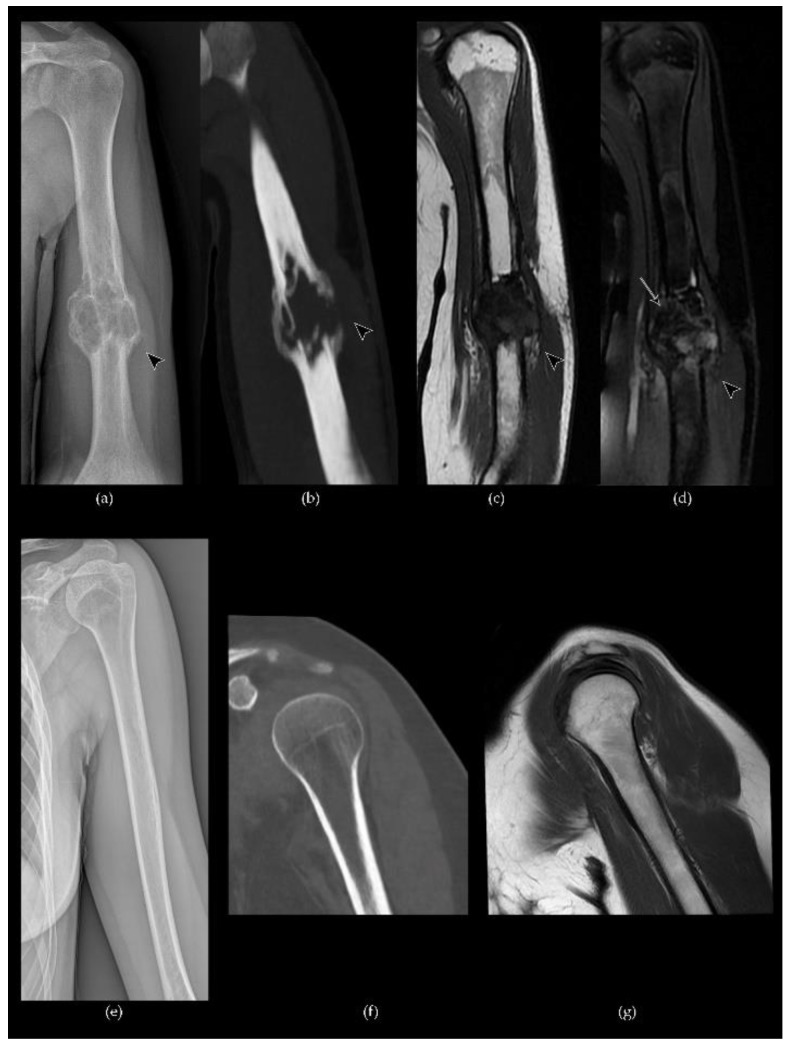
A case of GCTB of the left humerus diaphysis (**a**–**d**, arrowhead) compared to normal bone (**e**–**g**): (**a**) conventional radiography shows an osteolytic alteration of the bone with a pluri-chambered appearance in relation to the cystic components; (**b**) a coronal CT Maximum Intensity Projection (MIP) shows the erosion of the cortical bone with loss of the classic trabeculation and pathological fracture associated; (**c**) on a coronal T1-W FSE MR image, the lesion demonstrates inhomogeneous low signal intensity with a pathological involvement of the adjacent soft tissues; (**d**) a coronal fat-suppressed T2-W FSE MR image shows heterogenous signal intensity, with cystic components characterized by focal hyperintensity and hemosiderin deposits (arrow); (**e**–**g**) conventional radiography, CT and T1-W FSE MR images of normal bone findings.

**Figure 5:**
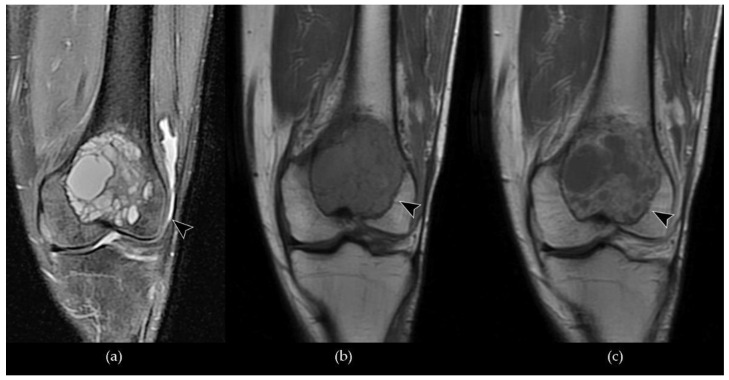
Coronal MRI of a GCTB of the distal metaepiphysis of the left femur (arrowhead) with associated joint effusion: (**a**) a fat-suppressed T2-W FSE scan shows a lesion with inhomogeneous signal hyperintensity in relation to the presence of multiple cystic components; (**b**) a T1-W FSE image shows non-uniform signal hypointensity; (**c**) a post-contrast T1-W FSE image demonstrates heterogeneous enhancement of the lesion, predominantly peripheral, and well discriminates the cystic and solid components.

Intravenous gadolinium administration usually shows heterogeneous enhancement of the lesion and permits the differentiation between the cystic and solid components, enabling a proper differential diagnosis with a primary aneurismal bone cyst [63]. MRI is also the method of choice to demonstrate tumor extension to the adjacent joint and soft tissue and serves as a pre-biopsy assessment to identify and demarcate the intralesional solid component [61] (Figure 4 and Figure 5).

In the last decade, radiomics has gained an increasingly important role as a valuable instrument for radiological and clinical practice [69,70]. Radiomics is a complex image analysis technique based on the extraction of quantitative metrics, called features, within medical images, defining regions of interest (ROI). It has found wide use in oncology, since features such as heterogeneity and shape have been correlated to the lesion’s biological behavior, so that, in combination with demographic, histologic, genomic or proteomic data, they can provide additional information about the tumor, potentially predicting clinical endpoints, such as survival and treatment response [71,72]. Consistent with this, radiomics can be seen as a useful tool for physicians in planning clinical trial settings specifically tailored to each patient in accordance with quantitative parameters and outcome data [73].

Radiomics has proven to offer a valuable contribution to GCTB pre-operative diagnosis, specifically aiding differential diagnosis, as reported in some studies by Ying et al. [74,75,76]. The authors validated a radiomics model on 120 pathologically confirmed sacral lesions, including primary sacral chordoma, sacral GCTB and metastatic tumor of sacrum, with the aim of helping in the determination of a preoperative differential diagnosis based on T2-W and contrast-enhanced T1-W MRI studies. The results identified 10 features associated in a statistically significant way to the histopathological diagnosis; the radiomics model based on a combination of fat-saturated T2-W and contrast-enhanced T1-W images, reached an AUC of 0.773 and an accuracy (ACC) of 0.711, suggesting that a texture analysis may assist physicians in diagnosis and possibly support the choice of a personalized treatment [74]. Similarly, in another study [76] on 137 patients, the authors tested different clinical-radiomic models based on CT scans and multiparametric MRI in distinguishing sacral chordoma and sacral GCTB before operation; in particular, the clinical-radiomic nomogram based on combined CT features achieved the highest AUC of 0.948 and an accuracy of 0.920. Recently, in a larger retrospective study involving 795 patients [75], the same group assessed the performance of non-parametric machine learning random forest radiomic models (RF-RMs) based on pre-operative CT imaging and clinical features in differentiating pelvic and sacral tumor types (metastasis, chordomas, GCTBs, chondrosarcomas, osteosarcomas, neurogenic tumors and Ewing’s sarcomas). The results proved that clinical-radiomic models built on statistically significant clinical features combined with CT radiomic features performed better than individual RMs and clinical models, providing valuable discriminatory performance in predicting pelvic and sacral tumor type (AUC = 0.928 and ACC = 0.877 in distinguishing GCTB from neurogenic tumor).

### 3.2. Preoperative Imaging Correlation with Staging and Prognosis

Historically, GCTB intermediate biological behavior has posed the problem of achieving correct diagnostic and therapeutic management; thus, several staging systems have been proposed over the years [19,77,78,79]. Enneking’s classification was based on a combination of radiological and histological criteria that identified four different progressive phases of the tumor, where the first three included a benign histology, while the fourth involved a sarcomatous lesion. The first stage, called “latent”, referred to an asymptomatic lesion confined by bone; the second stage, defined as “active”, was characterized by an active lesion on a bone scan associated with thinned bone cortex and frequent spontaneous fractures. The third stage was the “aggressive” one, characterized by a rapidly growing mass, causing cortical erosions and possible infiltration of adjacent soft tissues and metastases; the fourth stage, the “malignant”, was characterized by sarcomatous aggressive behavior [77,80]. Campanacci et al. [77,78] reduced the classification to three grades based on conventional radiography findings: grade one (latent) corresponded to a lesion with a well-defined margin, presence of sclerotic border and absence of cortical involvement. In grade two (active), the tumor had well-defined margins but no peripheral sclerosis, with thinned cortical bone; in the third grade, the lesion showed indistinct edges, soft tissue infiltration and erosions of the cortex.

However, the prognostic significance of these classifications has still not been clearly established yet, even though a higher rate of local recurrence in Campanacci grade three tumors has been observed compared with grade two tumors [19,78,81,82].

He et al. [83] conducted a prospective study on 48 GCTBs in the proximal tibia and distal femur treated with curettage in order to evaluate the role of preoperative imaging in predicting local recurrence. MRI cystic changes and adjacent soft tissue invasion were associated with a higher rate of local recurrence compared to the negative groups (*p* < 0.05). Cystic changes are an expression of liquefactive necrosis and intra-tumoral hemorrhage, probably due to an overexpression of vascular endothelial growth factor (VEGF), which plays an important role in osteoclast formation and resorptive activity, and it is usually found in advanced GCTBs. Cystic changes proved to be an independent risk factor for local recurrence (*p* < 0.05).

Similarly, He et al. [84] evaluated preoperative imaging features in 22 patients with GCTB recurrence after intralesional curettage with polymethylmethacrylate (PMMA) packing in order to identify the prognostic factors for local recurrence through an assessment of the preoperative imaging features of the tumor border. Among the preoperative CT and MR imaging features evaluated, the most relevant was the “paintbrush borders” sign, identified with the presence of penetrating irregular margins with a “paintbrush” appearance protruding toward the bone on an MRI, specifically in T1-weighted images. Patients with a “paintbrush borders” sign (21/22) had a significantly higher rate of local recurrence (71.43%) than patients without (21.88%), and it was identified as an independent prognostic factor for local recurrence (*p* < 0.05).

In following studies [85,86,87], the “paintbrush borders” sign and the cystic changes detected in a preoperative MRI of GCTBs treated with intralesional curettage were positively correlated with the expression of matrix metalloproteinase-9 (MMP-9) through an immunohistochemistry analysis (*p* < 0.05). Moreover, MMP-9 was also correlated with the expression of RANKL and VEGF (*p* < 0.05); however, VEGF, RANK and RANKL expression was not associated with the aforementioned preoperative MRI features of GCTB (*p* > 0.05) [86,87]. This may explain the molecular basis of the “paintbrush borders” sign; in fact, since MMP-9 stimulates bone resorption by giant cells, it is plausible that this MRI feature might indicate osteolytic destruction and invasion of the bone around the lesions [47,85,86,87]. Nevertheless, high expression levels of VEGF and MMP-9 have been correlated with aggressive GCTB biological behavior and a higher risk of local recurrence [88].

Zhou et al. [89] systematically investigated the prognostic value of preoperative CT images in 211 patients subject to extended curettage for GCTB, measuring tumor size, distance between the tumor edge and articular surface, thickness of the thinnest part of the cortex affected by the tumor and thickness of unaffected bone cortex. Statistical analyses demonstrated that the distance from the tumor edge to the articular surface < 2 mm, thickness of unaffected cortical bone around the tumor < 3 mm and patient age at surgery were independent risk factors associated with GCTB recurrence (*p* < 0.05, C-index 0.82 (CI, 0.76 to 0.88)).

In the era of computer-aided diagnosis tools based on machine learning, deep-learning convolutional neural networks (CNN) and their variants have been increasingly used in medical image pattern recognition as integrative instruments to aid radiologists in the correct diagnosis [90]. In this regard, the same group of He et al. [91] aimed to predict GCTB local recurrence after intralesional curettage using a deep CNN, evaluating pre-surgery MR images, whose results were compared with the same analysis performed by four musculoskeletal radiologists of at least 12 years’ experience. The accuracy of the CNN to predict GCTB prognosis (recurrent or non-recurrent) was higher than radiologists’ (78.6% versus 64.3%), as well as sensitivity (87.5% versus 58.3%), while specificity was similar for both (75.0%).

In this regard, Wang et al. [92] investigated the role of radiomics analysis on preoperative CT imaging in predicting early postoperative recurrence of 62 patients with spine GCTB. The AUC of the final prediction model using 10 features extracted was 0.78 with an accuracy of 89%, suggesting that the radiomics model has the potential to provide a personalized relapse risk assessment, on whose basis surgery, adjuvant treatments and postoperative follow-up should be assessed.

### 3.3. Post Surgical Imaging Evaluation

The complexity of GCTB management lies in the high recurrence rate after treatment that can vary from 2.5% to 45%, according to the type of surgical procedure and local presentation of the tumor [8]. Intralesional curettage is associated to a higher recurrence rate than wide surgical resection [83,93]. Therefore, post-treatment radiological follow-up plays a pivotal role in the early detection of loco-regional recurrence signs [60].

As stated by Wang et al. [94], imaging features associated with local recurrence are bone resorption around the graft bone or the polymethylmethacrylate (PMMA) cement, soft tissue mass formation and expansile destruction of bone and surrounding soft tissues.

In the case of local recurrence, follow-up radiographs after curettage and PMMA packing typically demonstrate increased lucency at the bone–cement interface that can be evaluated through cross-sectional CT imaging, which possibly enables appropriate biopsy planning [60,95,96].

On MR imaging, a bone recurrent tumor appears as a heterogeneous area of high signal on T2-weighted images and a low signal on T1-weighted images, with or without cystic components or aneurysmal bone cyst changes with the characteristic fluid–fluid levels indicating hemorrhagic areas [96,97]. In the case of curettage with cement, T2-weighted images demonstrate signal void of the cement and high signal of the adjacent proximal lesion [96].

Recurrence in soft tissues is rare compared to bone recurrence, and it usually occurs in the area close to the curettage site, most likely due to contamination during surgical treatment [93,98]. In this case, radiologic evaluation demonstrates a soft-tissue mass with inhomogeneous high-intermediate signal intensity on fat-suppressed T2-wieghted images [98]. Moreover, the recurrent lesion usually presents a peripheral rim of ossification, expression of metaplastic bone formation, which can be detected through CT imaging or plain radiography [93,98].

### 3.4. Post Denosumab Imaging Evaluation

Denosumab administration has proven to reduce GCTB size and progression by interfering with the activity of osteoclast-like cells, inducing consequent bone mineralization and intralesional bone density increase [99]. Particularly, it stimulates the reconstruction of a new peripheral osseous rim and the gradual ossification of the tumor, which is associated with a simultaneous reduction of the cystic and necrotic-hemorrhagic intralesional component, downstaging the lesion from aggressive to active or latent [25].

Therefore, imaging has an important role in the qualitative and quantitative evaluation of the response to denosumab treatment; however, to date, there are still no uniform imaging assessment criteria to properly evaluate the denosumab treatment response in GCTB.

**Figure 6:**
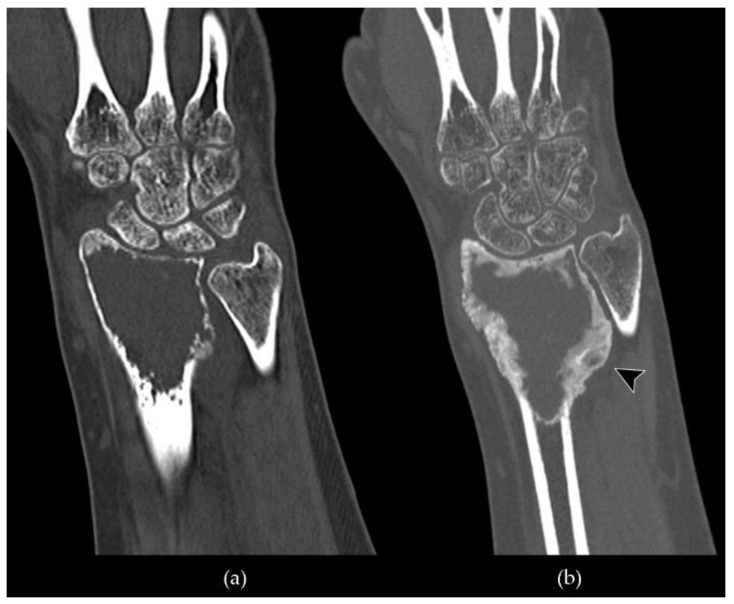
Coronal CT scan of a giant cell tumor of bone of the distal metaepiphysis of the radius treated with denosumab: (**a**) a pre-treatment image shows an osteolytic lesion with noticeable cortical thinning and endosteal scalloping; (**b**) a post-treatment image demonstrates peripherical osteosclerosis around the lesion with neocortex formation (arrowhead).

**Figure 7:**
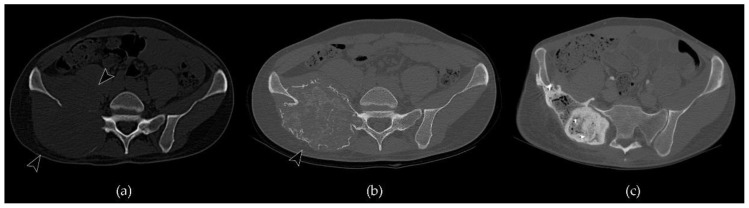
Axial CT scan of a case of GCTB of the sacrum and right iliac wing treated with denosumab and then subjected to surgical osteotomy: (**a**) a pre-treatment image shows an extensive osteolytic lesion, which extends to the adjacent soft tissues, involving the gluteal area and the pelvis (arrowheads); (**b**) a post-denosumab image demonstrates peripherical deposition of bone matrix with neocortex formation and intralesional osteosclerosis (arrowhead). A modest size reduction of the lesion can also be noted in relation to the shrinkage effect; (**c**) post-surgical evaluation highlights the outcomes of osteotomy in the right iliac wing filled with cement and two K-wires.

On plain radiography and CT evaluation, the main feature suggestive of a positive response to denosumab is represented by peripherical well-defined osteosclerosis around the lesion with neocortex formation; intralesional osteosclerosis can be identifiable after two weeks of treatment with different levels of internal matrix consolidation [100,101]. CT has the advantage of quantifying the density of the lesion in Hounsfield units, which represents an direct indicator of the sclerosis degree [100]. Denosumab has also been associated with tumor shrinkage effects, as proven by a quantitative assessment made on CT images by Yonezawa et al. [102] on four patients with spinal GCTB treated with denosumab before surgery (Figure 6 and Figure 7).

MRI is another valuable method in the assessment of GCTB after denosumab administration. A positive response is demonstrated by the reduction of tumor size and shrinkage of the cystic component, as well as a significant decrease in both the T1- and T2-weighted image signal intensity ratio [103].

The rationale of these findings is based on the inhibition of osteoclastic activity, which determines the reduction of the necrotic-inflammatory component (markedly hyperintense) and promotes tumor ossification or tumor replacement by intermixed bone tissue and fibroblast-like spindle cells, which are typically hypointense [101,104]. Changes in the intensity of post-contrast enhancement on T1- weighted images or in the mean ADC value of the solid part of the tumor did not prove to be statistically related to a positive treatment response [104].

In this regard, Furuta et al. [105] recently investigated the utility of intraoperative MRI for detection of residual tumors during surgical curettage in five patients with GCTB who received denosumab. The study demonstrated that iMRI was able to identify in all five cases suspected residual tumor tissue, confirmed by histopathology after additional curettage (detection rate was 100%), suggesting iMRI may represent a useful tool to promptly identify residual tumors and consequently prevent local recurrence.

Given the absence of a univocal system of standardization of radiological GCTB treatment response, Engellau et al. [99] compared tumor response to denosumab using Response Evaluation Criteria in Solid Tumors version 1.1 (RECIST), European Organization for Research and Treatment of Cancer response criteria (positron emission tomography (PET) scan criteria) and inverse Choi density/size (ICDS) criteria on CT, MRI and whole-body 18FDG-PET images. The proportions of patients with an objective tumor response were 35% per RECIST, 82% per PET scan criteria and 71% per ICDS criteria (size/density). RECIST criteria resulted as potentially insensitive in assessing the response to denosumab since they are purely size-based, while GCTB more frequently responds to denosumab with ossification rather than size reduction, and thus, ICDS provided better early indication of treatment response.

Campanacci et al. [106] recently assessed radiological responses to denosumab prior to surgery in 36 patients with GCTB, comparing Choi criteria based on size and tumor density and a newly described computerized tomography (CT) classification based on size, ossification of the tumor shell and internal ossification. In the study, the proportions of patients with a radiological tumor response were 89% per Choi criteria and 100% according to the new CT proposed classification, which proved to evaluate more accurately the response to denosumab.

Radiomics has proven to offer promising alternative solutions for the quantitative assessment of the GCTB response to denosumab, as demonstrated by Yi et al. [107] in a study aimed to compare CT radiomic features of GCTB before and after denosumab treatment, establishing their suitability in monitoring GCTB response to denosumab. Among the features evaluated, mean attenuation, standard deviation, entropy and skewness had significantly increased after treatment (*p* < 0.05), suggesting that CT histogram and textural features of GCTBs might aid disease monitoring during post-denosumab surveillance.

Recently, Chan et al. [108] proposed a similar study to determine denosumab response from a radiomics analysis of GCTB radiographs in 10 patients. Radiomics features were normalized based on intensity values from adjacent non-tumor bone; among the features extracted, mean intensity (*p* = 0.033) significantly increased while skewness (*p* = 0.028) significantly decreased after treatment, suggesting that radiomics analyses of plain radiographs can quantify time-dependent matrix mineralization and trabecular reconstitution that marks a positive response of GCTB to denosumab.

## 4. Conclusions

GCTB is classified as an intermediate malignant tumor due to its locally aggressive behavior, burdened by a high local recurrence rate. For this reason, imaging contribution is a fundamental aid for treatment planning and for identifying those characteristics that may be associated with prognosis. On preoperative MRI, features such as cystic changes, penetrating irregular margins and adjacent soft tissue invasion have been associated with a higher rate of local recurrence. Preoperative CT characteristics like distance from the articular surface < 2 mm and thickness of unaffected cortical bone < 3 mm, have also been reported as independent risk factors associated with recurrence. Local recurrence after surgical curettage typically demonstrates increased lucency at the bone–cement interface on CT imaging and the development of cystic components on MRI. A denosumab positive response is represented by peripherical well-defined osteosclerosis as well as inside the lesion. CT is able to quantify the density of the lesion in Hounsfield units, which represents a direct indicator of the sclerosis degree. In this sense, radiomic texture analysis has proven to offer promising alternative solutions for aiding pre-operative diagnosis and for the quantitative assessment of GCTB response to denosumab, both on conventional radiography and CT.

## Data Availability

No new data were created or analyzed in this study. Data sharing is not applicable to this article.
